# Prevalence of Silicone Lymphadenopathy in Women with Breast Implants: A single-center retrospective study

**DOI:** 10.1016/j.jpra.2025.01.016

**Published:** 2025-01-30

**Authors:** Juliënne A. Berben, Esther M. Heuts, Thiemo J.A. van Nijnatten, René R.W.J. van der Hulst

**Affiliations:** 1 Department of Plastic, Reconstructive, and Hand Surgery, Maastricht University Medical Center+, Maastricht, the Netherlands; 2 NUTRIM School of Nutrition and Translational Research in Metabolism, Maastricht University, Maastricht, the Netherlands; 3 Department of Surgery, Maastricht University Medical Center+, Maastricht, the Netherlands; 4 Department of Radiology and Nuclear Medicine, Maastricht University Medical Center+, Maastricht, the Netherlands; 5 GROW Research Institute for Oncology and Reproduction, Maastricht University, Maastricht, the Netherlands

**Keywords:** Silicone Lymphadenopathy, Silicone Breast Implants, Implant Rupture, Ultrasound, breast MRI, Siliconoma

## Abstract

**Introduction:**

Silicone lymphadenopathy (SLA) is a known finding after breast implant surgery. The prevalence of SLA is unknown and therefore its clinical implications are unclear. To make a statement about the clinical importance of SLA, more knowledge on its prevalence is necessary. This study aimed to provide details of SLA prevalence in a single-center.

**Methods:**

This single-center retrospective cohort collected all breast radiology reports from breast or axillary ultrasound (US) and breast MRI exams between 2010 and 2020. These reports were screened for the presence of implant rupture (IR) and/or SLA.

**Results:**

Overall, 1,217 women with silicone breast implants (SBIs) were included over 10 years. This resulted in 1,345 US and 900 MRI reports. In this cohort, 47 women (3.86%) had SLA with intact SBIs, 136 women (11.18) had IR, and 24 (1.97%) had SLA with IR. The sensitivity for IR on US and MRI were 76.2% and 91.7%, respectively. The specificity was 53.8% for IR on US and 66.7% on MRI. These calculations were based on the imaging results of patients whose implants were removed in the MUMC+.

**Conclusion:**

This retrospective cohort provides a single-center ten-year representation of diagnostic imaging of patients with breast implants. The prevalence of SLA in this cohort of women with breast implants is 5.83%. IR increases the risk of developing SLA; however, it can also occur in women with intact SBIs. To our knowledge, this is the first study to report on the prevalence of SLA in patients with SBIs.

## Introduction

Over 10 to 35 million breast implants have been placed worldwide.^1^^,^^2^ An estimated 3% of the Dutch female population has received silicone breast implants (SBIs) for reconstruction after breast cancer or breast augmentation.^3^ Known complications of breast implant surgery include surgical risks such as bleeding and infection and implant risks such as rupture, leakage, and silicone migration.^4^^,^^5^ The prevalence of implant rupture (IR) is up to 55% with a median rupture age of 10.8 years. Overall, 22% of the ruptured implants in the study by Brown et al. showed extracapsular spread of silicone.^6^^,^^7^ The 10-year implant survival was estimated to be between 83%-85% by Hölmich et al.^8^ The spread of silicone can cause local inflammatory foreign body reaction referred to as silicone granuloma, which will present as a hard palpable mass.^9^ A rarer complication of implant rupture is silicone lymphadenopathy (SLA). SLA can be an indication of IR but can also occur in the case of an intact implant. This is referred to as “silicone leakage” or “gel bleed,” where silicone particles migrate through the elastomer shell of the silicone implant.^10^

Although SLA is considered a rare complication, there are very few studies on the subject, with only 1 publication reporting an incidence of 13.6%.^11^^,^^12^ A recent review by Perez et al. showed that the existing evidence mostly comprises case reports.^13^ SLA occurs predominantly in the axillary lymph nodes (73%).^14^ However, it is also mentioned in internal mammary and mediastinal lymph nodes. 15 Case reports of cardiophrenic and paratracheal SLA have also been published.^16^^,^^17^ Although SLA does not require treatment unless symptomatic^11^, it can cause concerns when discovered while imaging, especially in patients with a previous history of breast cancer.^18^ In patients with recent breast cancer diagnosis and SLA, it is difficult to distinguish SLA from the malignant lymph nodes on imaging and, therefore, it poses challenges in the axillary surgical treatment strategy.^19^

To make a statement regarding the clinical importance and implications of SLA and possibly reassure breast cancer survivors, more knowledge on SLA prevalence is necessary. Therefore, this study aimed to provide details about SLA prevalence from a single center.

## Methods

### Study design

This single-center retrospective cohort study was performed at the Maastricht University Medical Center (MUMC+).

All breast or axillary ultrasound (US) and breast MRI reports within 10 years (2010-2020) were collected. Within these records, a search using implant-related keywords (Appendix A) was conducted to identify women with breast implants. To verify the presence of SLA, these reports were searched for SLA-related keywords (Appendix A). Additionally, to identify women with IR, reports of all women with breast implants were searched for IR-related keywords (Appendix A). The reports mentioning SLA or IR were assessed by 1 reviewer (JB) to select patients with SLA and/or IR. In case of ambiguities, a breast radiologist (TvN) was consulted. Identical ultrasound reports of the left and right breast or axilla were considered as a single ultrasound report.

### Mismatched results

If a patient underwent breast and/or axillary US and breast MRI exams, but the results did not align, the presence of SLA was confirmed based on whether SLA was diagnosed using any form of imaging. The presence of IR was based on MRI exam results, as breast MRI is believed to be more sensitive for detecting IR.^20^ However, in case the US exam was carried out 12 months or more after the last breast MRI exam, the US exam result was given preference.

This study was approved by the Medical Ethics Review Committee of AZM/Maastricht University (METC 2022-3244).

### Statistical analysis

Descriptive statistics were used to determine the prevalence of SLA and IR in this population and to describe patient demographics and clinical characteristics. All analyses were performed using SPSS statistics version 29.

## Results

**Figure 1:**
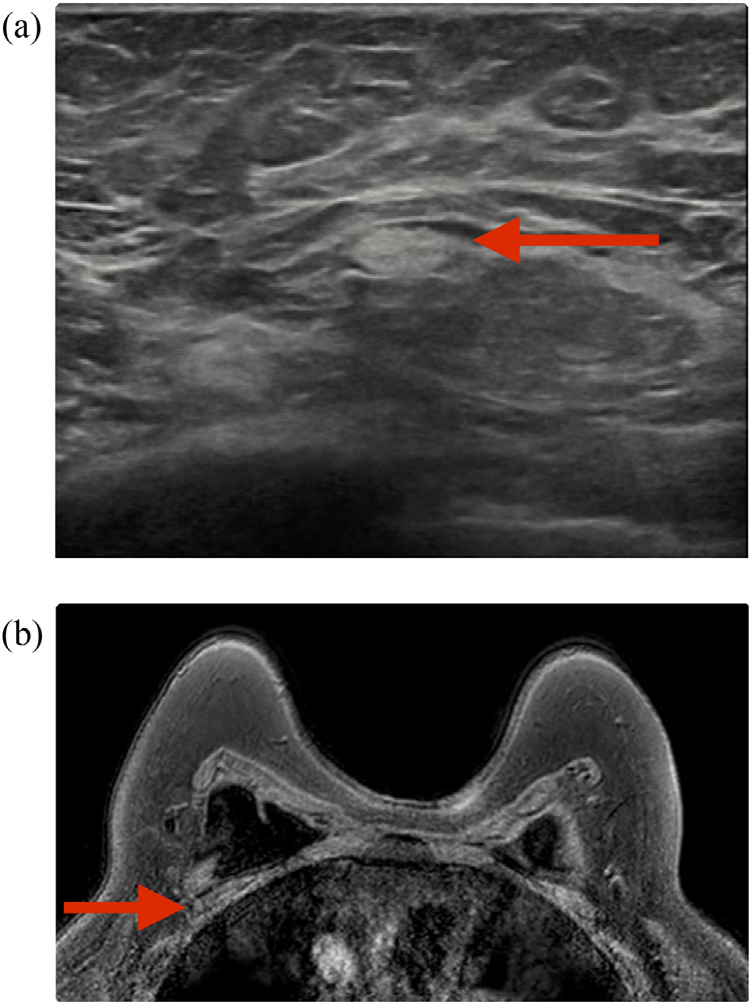
Imaging of axillary US and breast MRI exam of a patient with SLA.

**Figure 2:**
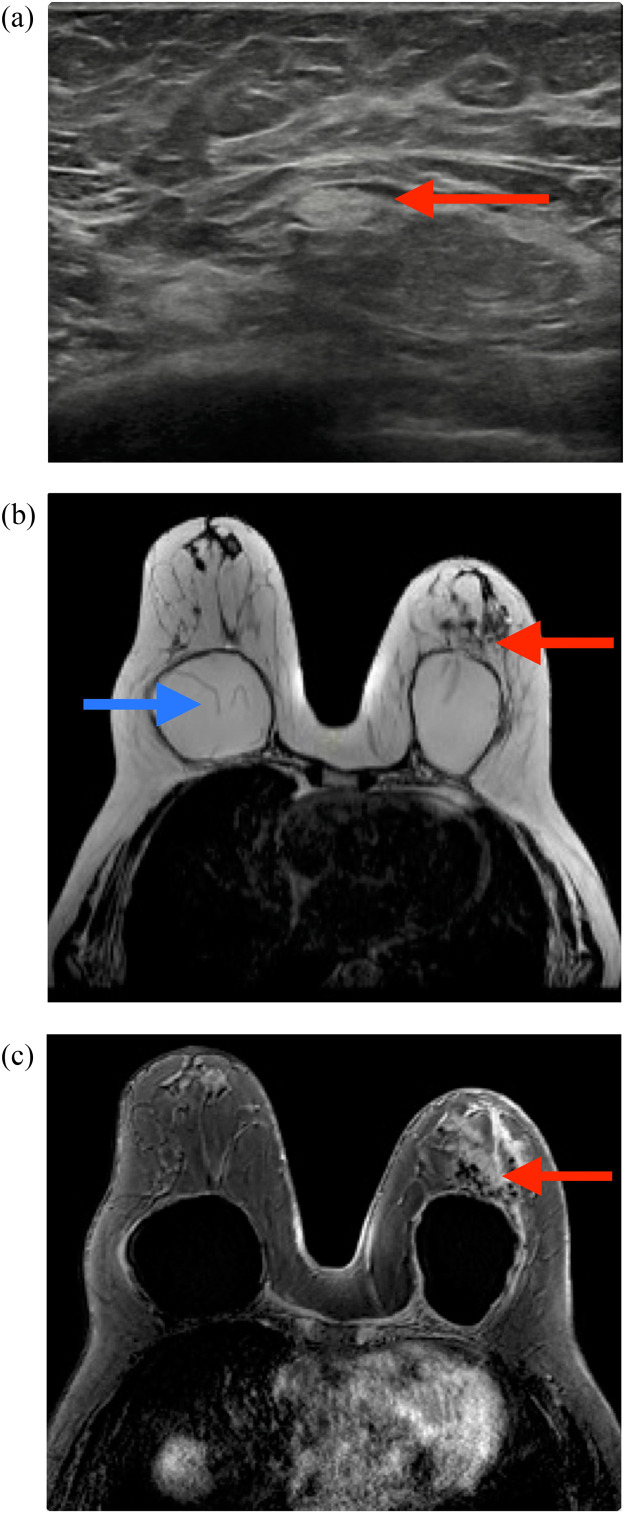
Imaging of breast US and MRI exam of a patient with SBI rupture

This single-center retrospective cohort included 1,217 patients with SBIs. Two hundred and twenty-six women had imaging reports that mentioned either SLA, SLA with IR, or IR. Their mean age at the time of diagnosis was 25.9 ±12.4 years. Among the 226 women, 36 (15.9%) had SBIs for reconstructive purposes. The baseline demographics are presented in Table 1. Figures 1 and 2 show examples of SLA and IR in US and breast MRI exams.

**Table 1:** Demographics and clinical characteristics.

	n = 226
*Age at time of diagnosis (years)*	25.9 ± 12.4
*Indication for SBI (n, %)*	
*Reconstruction*	36 (15.9)
*Augmentation*	184 (81.4)
*Currently no implants*	5 (2.2)
*Unknown*	1 (0.4)
*Type of breast implant (n, %)*	
*Silicone*	173 (76.5)
*Saline*	33 (14.6)
*Unknown*	20 (8.8)
*First implants (n, %)*	127 (56.2)
*Total number of breast MRI exams (n = 165)*	236
*Mean number of breast MRI exams per patient*	1.4 ± 1.4 (1-12)
Total number of breast ultrasound exams (n = 171)	293
*Mean number of breast ultrasound exams per patient*	1.7 ± 1.2 (1-12)
*Both breast MRI and breast ultrasound exams* Silicone lymphadenopathy (n, %*) Silicone lymphadenopathy and Implant Rupture (n, %*) Implant rupture (n, %*)	110 47 (3.9) 24 (2.0) 136 (11.2)

*Share of total study population with SBIs (1,217).

An example case of a 48-year-old woman with bilateral breast implants. Ultrasound of the right axilla (a) showing snowstorm appearance (red arrows) in the axillary lymph nodes, suggesting SLA. This was confirmed using breast MRI exam showing low signal intensity on the silicon suppressive sequence (red arrow) (b). MRI, magnetic resonance imaging; SLA, Silicone lymphadenopathy.

An example case of a 72-year-old woman with bilateral breast implants. Breast ultrasound (a) shows a snowstorm appearance (red arrow) in the retro areolar region of the left breast, highly suggestive of extracapsular rupture of the prepectoral breast implant. Breast MRI exam confirmed extracapsular rupture of the breast implant in the left breast (red arrows) on the T2W (b) and silicone suppressive sequences, respectively (c). Note in the right breast a “linguini sign” (blue arrow), representing an intracapsular rupture (b). MRI, magnetic resonance imaging; SBI, silicone breast implant.

**Figure 3:**
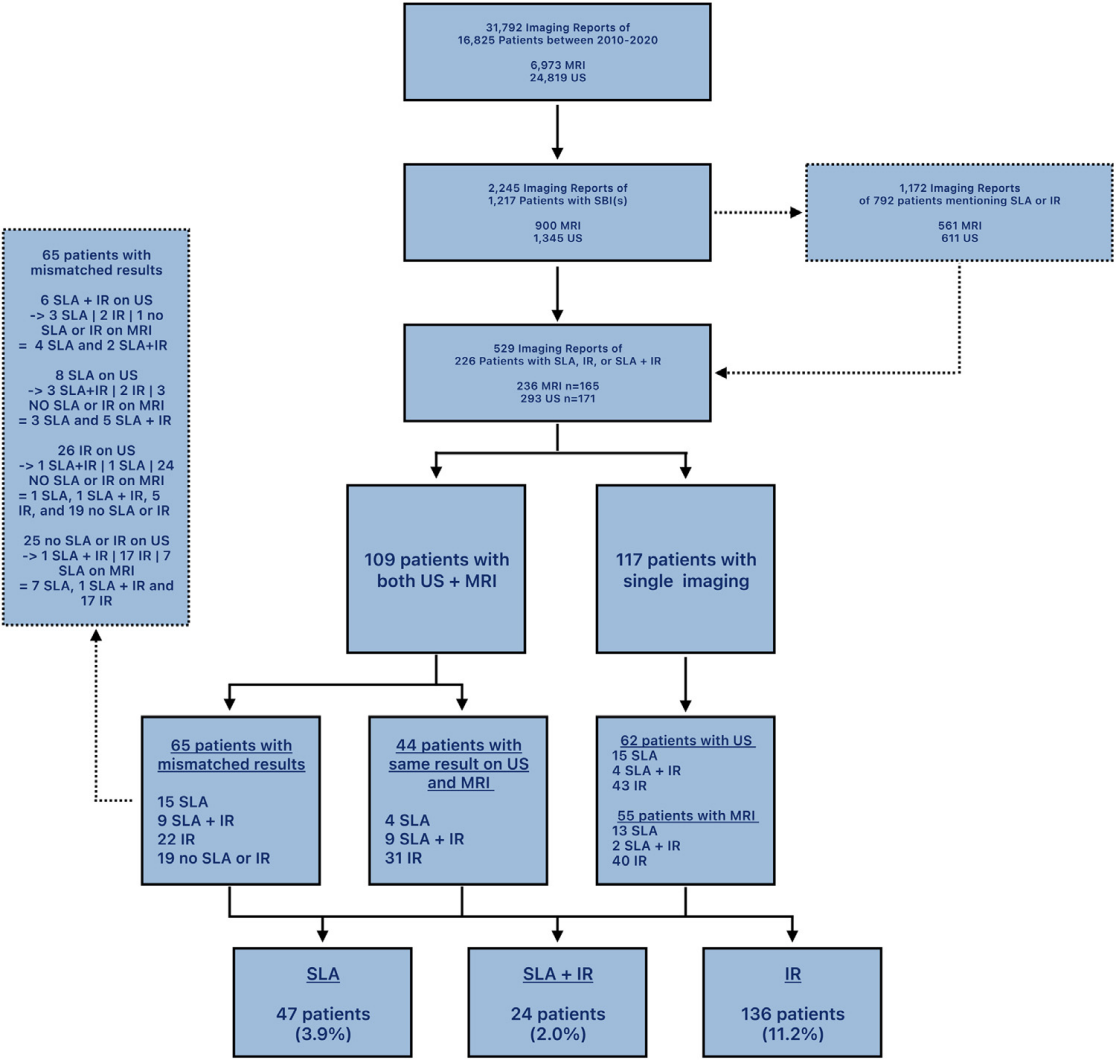
Flowchart depicting data selection.

Twenty-four (1.97%) women presented with SLA with IR, 47 (3.86%) women had SLA without IR, 136 (11.18%) women had IR without SLA, and 19 women did not have SLA or IR after additional imaging. Women with SLA in the whole group of women with intact SBIs was 4.4%. Women with SLA and IR was 15%. The allocation of all women to their diagnosis is specified in Figure 3.

Among the 47 women with intact implants and SLA, 17 (36.2%) had no previous SBI or previous SBIs replaced after rupture, indicating that silicone spread by gel bleed of the intact SBI. Information on SBI history was missing in 10 (21.3%) of these women, with SLA and intact implants. Table 2 presents all implant-related outcomes.

**Table 2:** Implant-related outcomes.

	SLA (n = 47)	SLA + IR (n = 24)	IR (n = 136)
*Confirmed on (n, %)*			
*Ultrasound*	19 (40.4)	6 (25.0)	48 (35.3)
*Breast MRI*	24 (51.1)	9 (37.5)	57 (41.9)
*Both*	4 (8.5)	9 (37.5)	31 (22.8)
*Explantation of implants (n, %)* *Yes* *Unknown*	16 (34.0) 9 (19.1)	20 (83.3) 2 (8.3)	96 (70.6) 33 (24.3)
*Implant rupture confirmed during surgery (n, %)* *Yes* *Unknown*	2 (4.3) 36 (76.6)	12 (50.0) 10 (41.7)	46 (33.8) 82 (60.3)
*First time implant (n, %)* *Yes* *Unknown*	8 (16.3) 3 (6.1)	14 (58.3) 0	94 (69.1) 17 (12.5)
Previous SBI rupture (n, %*) *Yes* *Unknown* *Implant age (mean, SD) (years)* *Type of implant* *Silicone* *Saline* *Unknown*	23 (59.0) 7 (17.9) 9.0 ± 8.6 40 (85.1) 2 (4.3) 5 (10.6)	5 (50.0) 5 (50.0) 16.0 ± 10.1 23 (95.8) 1 (4.2) 0	9 (21.4) 23 (54.8) 17.1 ± 9.5 97 (71.3) 26 (19.1) 13 (9.6)

*Share of women with a previous implant.

Sixty-five women had mismatched results on the US and breast MRI exam. Among these, 32 IRs were detected on the US exam, among whom 29 were not detected on the breast MRI exam. Alternately, 23 IRs that were present on breast MRI were not detected in the US exam. As mentioned above, this resulted in 19 women with no diagnosis of SLA or IR. Figure 3 presents an overview of these results in a flowchart.

One hundred and forty women underwent SBI explantation. Among them, 116 women had a diagnosis of IR or SLA + IR on imaging. Only 79 women underwent explantation in the hospital where these data were collected and the status of the SBI during surgery was reported. Among the 79 patients, 68 had a diagnosis of IR or SLA + IR on imaging and IR was the indication for explantation. One woman had IR on her US exam, but the explantation surgery was postponed due to cardiac complications. However, IR was later confirmed by silicone leakage from her sternal wound. Among the 68 women with suspected IR on imaging, 59 cases of IR were confirmed during surgery. The sensitivity and specificity for IR on breast MRI and US were calculated using these data (Appendix B). The sensitivity was 76.2% and 91.7%, respectively, for US and breast MRI. The specificity for IR on US was 53.8% and 66.7% for breast MRI.

## Discussion

This retrospective cohort provides a single-center ten-year representation of breast imaging in patients with breast implants. The main goal of this study was to find the prevalence of SLA in this cohort. In total 71 (5.8%) women presented with SLA (with or without IR) on imaging.

It is often mentioned that SLA is a rare condition. SLA can occur in women with IR and women with intact SBIs. Apparently, the occurrence of SLA is not necessarily related to the presence of IR. However, with IR the risk of developing SLA appears to be elevated. As 15% of all women with IR had SLA and only 4.4% of all women had intact SBIs. The results of the cohort study by Rosenthal et al. also indicated an interaction between IR and SLA. 12 Among the 47 (3.9% of the total cohort) women with intact implants and SLA, 17 (1.4%) women, without previous implant or known rupture of a previous implant, had SLA. This indicates that gel bleed or silicone spread can also cause SLA because it was certain in these cases that previous IR did not cause SLA. However, when concentrating on the percentage of women with SLA within the group of all women with ruptured implants (SLA+IR and IR in Figure 2), the occurrence of SLA appears to be more common (24 women (15.0%) out of 160 women with IR).

Symptoms of SLA can be diverse, ranging from asymptomatic to burdensome and local or systemic complaints. Perez et al. reported a total of 279 cases of SLA in the current literature in which 55% had nontender lymphadenopathy or no symptoms at all.^13^ When a patient presents with a palpable mass, diagnostic imaging and possibly a biopsy will follow. Although biopsy is a minor procedure, it could lead to worry and anxiety in the patient. However, as SLA often appears with no or few symptoms, it can also be an incidental finding during imaging for other indications. First, this could mean that our data show an underreported prevalence. Second, in asymptomatic SLA, the process could be stressful in another way. One of the imaging modalities where asymptomatic SLA could be found is axillary US during breast cancer screening or follow-up. In the case of breast reconstruction, lymphatic drainage might be altered due to previous breast surgery. Therefore, SLA could be identified in unexpected places such as the contralateral axilla. When a patient is scheduled for PET-CT for staging purposes, SLA can be present as a false positive finding. In PET-CT exams, the F-fluorodeoxyglucose (FDG) uptake in SLA can cause increased metabolism in a lymph node indicating the potential to be malignant.^21^^,^^22^ In both cases, a biopsy has to be performed, considering the accompanying risks and worry. Therefore, SLA can cause distress, overdiagnosis, and diagnostic dilemmas, especially in patients with cancer history.^23^^,^^24^

Apart from the little knowledge on SLA occurrence, there is also no clarity on its clinical implications. A part of the population with SBIs that attributes their various systemic symptoms, such as fatigue, myalgia, night sweats, and several cognitive symptoms, to their implants. This syndrome is referred to as breast implant illness (BII). Although the exact pathophysiology is unclear, it is a hypothesized to be caused by an immune response to silicone spread.^25^ As SLA is caused by silicone spread, it could be an indicator of BII. The prevalence of BII-related symptoms varies widely between published studies (37.4%-65.0%) and these outcomes are most likely to be affected by selection bias.^26^^,^^27^ The percentage of women having their SBI removed because of BII-related symptoms is as low as 0.1%.^28^ Additionally, no literature is available on SLA prevalence in women reporting these symptoms. However, 10% of the women with SLA report systemic symptoms.^13^ Therefore, more research is needed to determine whether SLA is a predictor for BII.

Breast MRI is considered the most accurate breast imaging technique used to determine the presence of IR. National and international guidelines state that breast MRI is the recommended diagnostic tool for SBI imaging.^29^ According to the literature and our results, in addition to the higher costs and patient burden, breast MRI is less sensitive for detecting SLA than US but is more sensitive for IR.^30^^,^^31^ Although breast MRI is the gold standard for post-implant follow-up, compliance with the FDA recommendations is low as only 37.8% of plastic surgeons follow the FDA guidelines.^29^ The hospital from which these data were collected more often uses US as their diagnostic tool as it is more accessible and patient-friendly. An additional advantage is that SLA is more likely to be detected during US exam. In case of inconclusive results on IR, a breast MRI exam will subsequently be performed. Therefore, we suggest that US is a good option for post-implant follow-up. However, some patients in this study might have only had a breast MRI exam; therefore, the occurrence of SLA could have been underestimated. Contrastingly, some cases of IR were missed or over-diagnosed on the US exam. The sensitivity (76.2%) and specificity (53.8%) of US exams in this study confirm that breast MRI is superior for detecting IR. The results of the sensitivity of US are similar to that in literature (76.2 % vs. 73.7%). However, the specificity is much lower (53.8% vs. 87.8%).^32^ As this study was not specifically designed to calculate the accuracy of US and breast MRI for IR or SLA, the population on which these results were based is quite small. Therefore, no strong conclusions can be drawn from these outcomes. However, as mentioned, in case of inconclusive results on US, a breast MRI can be executed, and US is the more reliable and accessible tool for diagnosing SLA and IR.

This study had some limitations, including its retrospective single-center design. The US and breast MRI exams had several indications and SLA was often an incidental finding. The imaging reports were not always clear or conclusive on the matter, as not all radiologists provided a definitive answer on the presence of SLA. A secondary limitation was the inability to collect additional patient information such as implant age or the confirmation of IR during surgery. As the replacement of SBIs during explantation is often not reimbursed, such instance occur in private practices. However, by using retrospective data, imaging reports for a period of 10 years could be included, resulting in a large dataset. As no previous studies have researched SLA prevalence, we believe that the large amount of data can represent a baseline prevalence of SLA in women with SBIs.

Given that a considerable number of the women (5.8%) in this cohort developed SLA, it is relevant to research the clinical impact of SLA and analyze which of these women have symptoms. As systemic complaints related to BII might be caused by silicone spread, SLA could be a predictor for these complaints.^33^ Future studies should explore the risk of SLA in relation to IR and consequences of silicone spread such as systemic symptoms.

## Conclusion

This is one of the first studies reporting the prevalence of SLA in women with SBI. With a prevalence of 5.8% in this cohort, it can still be suggested that SLA is a rare complication of SBIs. Furthermore, 13.1% of women in this cohort had ruptured SBI. Although SLA also occurs in the patients with intact implants, suggesting gel bleed or silicone spread, IR appears to substantially increase the risk of developing SLA.

Although this study did not aim to research the sensitivity and specificity of US and breast MRI for SLA and IR, it could be suggested that US is a good option for breast implant follow-up. Especially, when considering lower costs and patient-friendliness. In case of inconclusiveness, a breast MRI can always be subsequently executed.

## Ethical approval

Ethical approval was given by the 10.13039/501100001835Medical Ethics Review Committee of AZM/Maastricht University (METC 2022–3244).

## Funding

The authors received no financial support for the research, authorship, and publication of this article. The authors also declare that no additional funding from public or private commercial or noncommercial entities will be received within the past 36 months.

## Declaration of competing interest

The authors declared no potential conflicts of interest with respect to the research, authorship, and publication of this article.

## Figures and Tables

**Table:** 

Implant related	IR related	SLA related
Breast prosthesis	Leakage	Silicone lymphadenopathy
Breast prosthesis (different spelling)	Rupture	Silicone lymphadenitis
Mamma-augmentation	Implant rupture	Silicone(adjective) lymphadenitis
(mamma)augmentation	Implant leakage	Silicone axillary
Augmentation		Extracapsular rupture
Breast Implant		Siliconoma
Implant		Silicone adenitis
Prosthesis		Silicone (adjuctive) adenitis
Silicone		Adenitis
Silicone(adjective)		Deposits
		Lymphadenitis
		Snowstorm sign
		Snowstorm sign (different spelling)
		Snowstorm
		Snowstorm appearance
		Lymphadenopathy

**Table:** 

	IR confirmed during surgery	No IR during surgery	Total
IR on breast MRI	44	6	50
No IR on breast MRI	4	12	16
Total	48	18	
IR on US	32	6	38
No IR on US	10	7	17
Total	42	13	

## Appendix A

Search terms imaging reports

## Appendix B

Numbers used for MRI and US sensitivity and specificity analyses
